# G4-quadruplex-binding proteins: review and insights into selectivity

**DOI:** 10.1007/s12551-022-00952-8

**Published:** 2022-04-20

**Authors:** Vanessa Meier-Stephenson

**Affiliations:** 1 Department of Medicine, Division of Infectious Diseases, University of Alberta, Edmonton, AB Canada; 2 Department of Medical Microbiology and Immunology, University of Alberta, Edmonton, AB Canada; 3 Li Ka Shing Institute of Virology, University of Alberta, Edmonton, AB Canada

**Keywords:** G4-quadruplexes, Quadruplex-binding proteins, DNA–protein interactions, RNA–protein interactions

## Abstract

There are over 700,000 putative G4-quadruplexes (G4Qs) in the human genome, found largely in promoter regions, telomeres, and other regions of high regulation. Growing evidence links their presence to functionality in various cellular processes, where cellular proteins interact with them, either stabilizing and/or anchoring upon them, or unwinding them to allow a process to proceed. Interest in understanding and manipulating the plethora of processes regulated by these G4Qs has spawned a new area of small-molecule binder development, with attempts to mimic and block the associated G4-binding protein (G4BP). Despite the growing interest and focus on these G4Qs, there is limited data (in particular, high-resolution structural information), on the nature of these G4Q-G4BP interactions and what makes a G4BP selective to certain G4Qs, if in fact they are at all. This review summarizes the current literature on G4BPs with regards to their interactions with G4Qs, providing groupings for binding mode, drawing conclusions around commonalities and highlighting information on specific interactions where available.

## Introduction

**Fig. 1:**
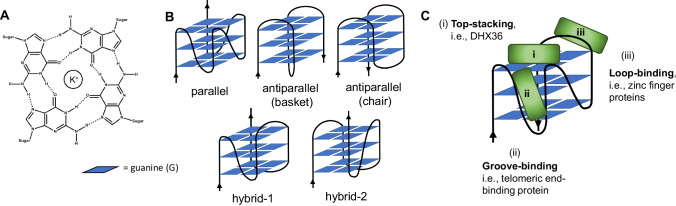
Schematic of G4-quadruplexes (G4Qs), showing the G-tetrad’s planar orientation (**A**), formed by Hoogsteen bonds and stabilized by a metal ion, typically potassium (K +), which can stack upon one another in various orientations (**B**). These structures interact with various cellular proteins, which may bind in a number of different manners (**C**), including top-stacking (i), groove-binding (ii), and loop-binding (iii)

G4-quadruplexes (G4Q) are guanine-rich secondary nucleic acid structures that form from stacks of the planar orientation of four guanosine residues held together by Hoogsteen bonds and stabilized by metal ions, typically K + ions (Fig. 1). The nucleic acid sequence that has the potential to form these structures is typically described in the formula *G*_*x*_*N*_*y*_*G*_*x*_*N*_*y*_*G*_*x*_*N*_*y*_*G*_*x*_, where *x* ≥ 2 guanosine residues and *y* is 1–7 nucleotides (N). They can occur in both RNA and DNA, and intramolecularly from a single strand of nucleic acid, or with multiple, typically 2 or 4 strands. Arrangements can vary in the nature of their stacking, forming parallel, antiparallel, and various hybrid formations, and while DNA G4Qs in nature can display a variety of these forms, RNA structures tend to be largely parallel (Zhang et al. 2010). Throughout the human genome, there are estimates of > 700,000 potential G4Q sequences (Hansel-Hertsch et al. 2017). Interestingly, these sequences show a high predominance in promoter regions, telomeres, and untranslated regions in mRNA (Hansel-Hertsch et al. 2017; Huppert and Balasubramanian 2005; Rhodes and Lipps 2015; Rigo et al. 2017). They are also found in the promoter regions and untranslated terminal regions (UTRs) of viruses (Fleming et al. 2016; Frasson et al. 2020; Lavezzo et al. 2018; Meier-Stephenson et al. 2021; Metifiot et al. 2014; Perrone et al. 2017). Their cross-species presence at these key regulation areas suggests a likely functional role in the DNA/RNA processing. Indeed, a growing body of evidence clearly links key cellular functions with these G4Q structures, in including transcription, translation, immunoglobulin class switch, and genome stabilization (Da Ros et al. 2021; Dalloul et al. 2018; Lerner et al. 2020; Siddiqui-Jain et al. 2002b; Wolfe et al. 2014). G4Qs have therefore become of great interest to the drug development realm for being able to target the respective downstream processes directed by these G4Qs (Asamitsu et al. 2019; Balasubramanian et al. 2011; McLuckie et al. 2013; Ohnmacht et al. 2015; Tauchi et al. 2006; Xu et al. 2017).

Current G4Q small-molecule binders and dyes appear to either sit atop the G4Q barrel or intercalate with the common G4Q core (Amato et al. 2018, 2017; Giancola and Pagano 2013; Phan et al. 2005; Sun et al. 2019). While varying affinities have been found for such molecules, there remains the challenge of improving selectivity to minimize off-target effects in the host cell. The very nature and selectivity of cellular proteins that interact with G4Qs imply that selectivity must be attainable. Determining the features of G4Q-binding proteins will be helpful in eliciting the details necessary to rationally design selective binders.

This review aims to assess the binding interactions of known G4-binding proteins (G4BPs) with their respective G4-quadruplexes, highlighting the commonalities in their interaction and subsequently features that would appear to make them selective.

## G4-quadruplexes (G4Qs): form and stability

G4Q formation kinetics and stability remain a topic of much research (Grün and Schwalbe 2022; Laouer et al. 2021; Lemkul 2019; Nguyen et al. 2020; Robinson et al. 2021; Rocca et al. 2020; Spiegel et al. 2020; Wu et al. 2020). While readily formed in vitro with temperature manipulations, the conditions under which G4Qs form in vivo are incompletely understood. The conformational energy landscapes are presumed to have many potential folding intermediates with favorable minima en route to the quadruplex form (Grün and Schwalbe 2022; Rocca et al. 2020). Once formed, the thermodynamic stability of the structure (of an intramolecular three-tetrad G4Q, for this example) is approximately as stable as the helical duplex DNA of similar length (Lane et al. 2008). The conformational forms a G4Q can assume (Fig. 1B) have been shown to influence thermodynamic stability, and while predominant forms are likely for certain G-rich sequences, there are also examples of mixed forms occurring from the same sequence (Chen and Yang 2012; Hatzakis et al. 2010; Lim et al. 2010). Even a shift along a longer G-rich stretch that changes only loop length can also influence G4Q stability (Hatzakis et al. 2010). Collectively, this suggests on ongoing flux and equilibrium among forms, rather than a fixed state. In comparing RNA and DNA G4Qs, there appears to be slightly greater thermodynamic stability of the RNA G4Q versus its DNA counterpart, likely owing to the additional hydrogen-bonding network enabled by the ribose hydroxyl groups, but always within the same order of magnitude (Arora and Maiti 2009; Joachimi et al. 2009; Zhang et al. 2010).

Formation and degradation of G4Qs have been shown in vitro to be dependent on a number of intrinsic factors including loop length, leading nucleic acids and incorporated ions (Bhattacharyya et al. 2016; Chen et al. 2021; Guédin et al. 2010; Hazel et al. 2004; Piazza et al. 2015). While it is possible that in vivo G4Qs are influenced by the same factors, there is the of course the additional interplay with many cellular proteins, namely, G4-binding proteins whose roles and influences are still being defined.

## G4-binding proteins (G4BPs)

There are several ways in which to categorize G4BP: one of which is by their influence on the G4s with which they interact, i.e., stabilizing versus destabilizing, but while there are a plethora of studies describing G4Q-G4BP interactions, the functional verdict remains to be determined for many pairings (Sun et al. 2019). Thus, this review has been organized by sites of binding, namely, G4BPs targeting telomeric, promoter, or RNA G4Qs. While the action of a G4BP may again be varied among groupings, the nature of the initial physical interactions is presumed to have commonalities and is so described where able.

### A preliminary note on G4BP-G4Q-binding modes

There are limited high-resolution structures of G4BPs interacting with G4Qs available, but some generalizations appear to be evolving, as will hopefully become apparent in this review. To highlight these binding modes up front for purposes of providing examples below, binding can occur at the following sites: (i) top-stacking with the upper G-quartets (i.e., atop the G4Q “barrel”), (ii) groove-binding (i.e., between the spaces of the loops), (iii) loop-binding (i.e., binding with the protruding loop nucleotides alone), or a combination of those modes (Fig. 1C). While only one to two concrete examples exist for each, others may become apparent as further biophysical and structural data become available supporting a particular mode. It is also possible that similar protein functionalities may support a particular binding mode (i.e., helicases may act through a stabilizing top-stacking interaction).

## G4BPs targeting telomeres

One of the first G4Q-forming sequences identified was in the human telomere (Wang and Patel 1993). Telomeres are the terminal segments of chromosomal DNA and have a highly G-rich 3′-overhang region containing hexameric repeats of TTAGGG (Brázda et al. 2014; O'Sullivan and Karlseder 2010). Functionally, telomeres play a critical role in genome stability, degrading with each cycle of replication. They are maintained by a number of proteins to ensure the process occurs in a well-regulated manner. Many of these proteins have been identified as G4Q-binding proteins, acting on the telomeric G4Qs in various ways, anchoring, degrading, stabilizing (Brázda et al. 2014). Some of these proteins known to bind the G4Qs in this region are Protection of telomeres 1 (POT1) protein, Replication protein A (RPA), human CTC1–STN1–TEN1 (CST), Breast cancer type 1 susceptibility protein (BRCA1), heterogeneous nuclear ribonucleoprotein A1 (hnRNP A1), Bloom and Werner’s syndrome proteins, and Preimplantation factor-1 (Pif1).

### Protection of telomeres 1 (POT1)

**Fig. 2:**
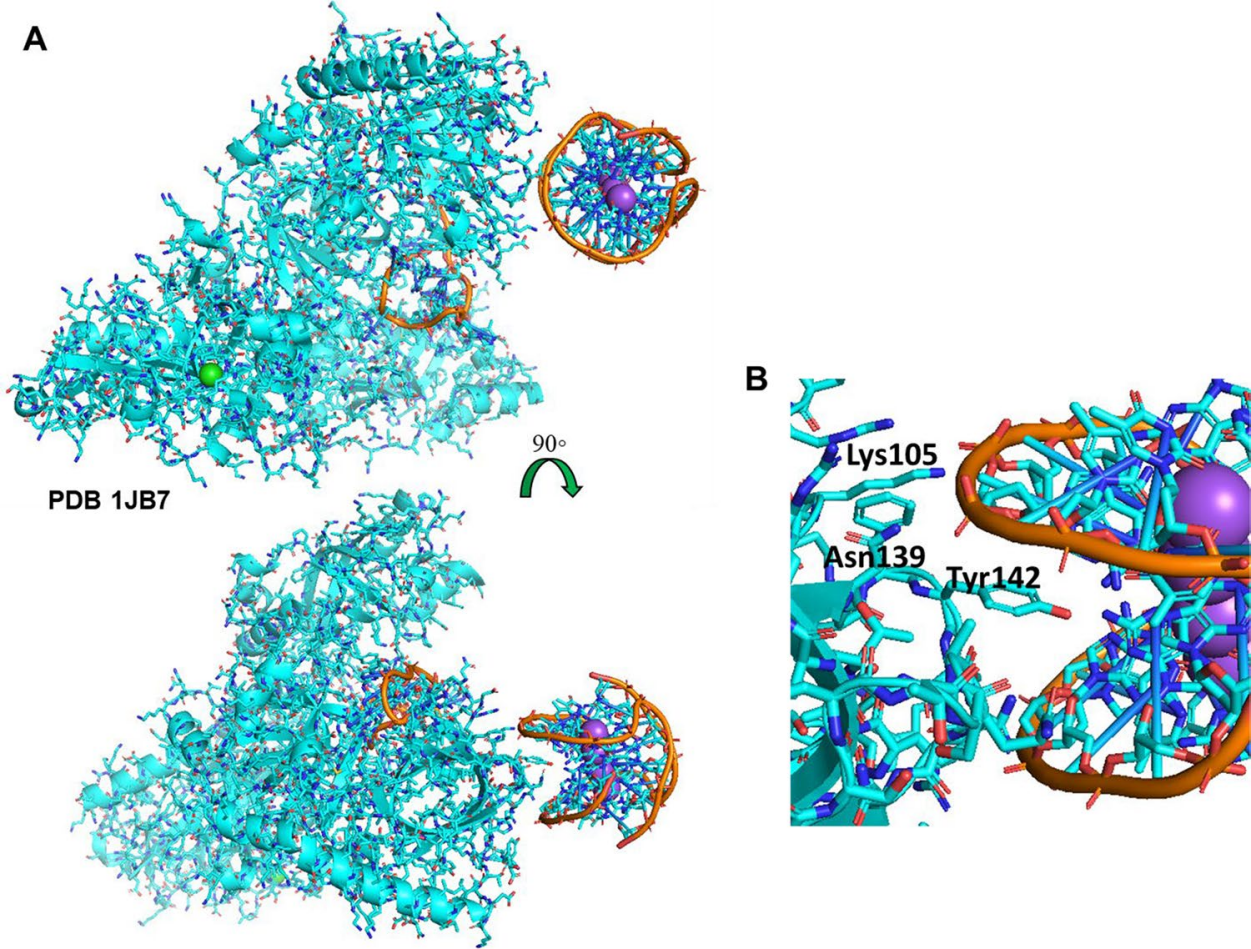
Example of groove-binding mode—telomeric end-binding protein of *Oxytricha nova* (OnTEBP), a protozoan analogue of human POT1 protein (PDB 1JB7) broad-view (**A**) and close-up (**B**), showing the Tyr142 in proximity to several of the G4Q guanosines and residues Lys105 and Asn139 nearer to the phosphate backbone facilitating H-bonding opportunities

POT1 protein is one of the six members of the telomere shelterin complex, which collectively prevent the telomeric overhang from being recognized as chromosomal damage (O'Sullivan and Karlseder 2010). Studies using single-molecule fluorescence resonance energy transfer (smFRET) were able to show that a monomer of POT1 binds the G4Q specifically, approaching it from the 3′-5′ direction, initiating unfolding of the G4Q (Hwang et al. 2012). It is subsequently followed by binding of a second monomer to complete the process in four sequential steps with the two monomers. Upon binding of the enhancer protein, tripeptidyl-peptidase 1 (TPP1), the POT1-TPP1 complex is thought to promote a sliding activity that unfolds then refolds the G-quadruplex(Hwang et al. 2012). Considered analogous to the POT1-TPP1 complex is the telomeric end binding protein in *Oxytricha nova* (Xin et al. 2007), and its NMR-derived structure is highlighted in Fig. 2 (Horvath and Schultz 2001). The crystal structure contains both an unwound strand of telomeric DNA and an intact G4Q at the border of the crystals’ unit cell. The G4Q, as the authors also highlight, appears to be interacting only minimally and *may* actually be an artifact of crystal packing, but several residues do appear to have the potential, including Lys105 and Asn139 interacting with the phosphate backbone of G4Q while Tyr142 reaches into the groove to interact with several of the guanosines (Fig. 2B) (Horvath and Schultz 2001). Interestingly, the POT1 G4Q binding has been shown to be selective to antiparallel G4Qs, while parallel G4Qs are unaffected (Ray et al. 2014). If in fact groove-binding is its main mode of interaction with a G4Q, this could be supported by components of steric hindrance among the various G4Q folding forms (examples in Fig. 1).

### Replication protein A (RPA)

RPA protein is the most abundant single-stranded DNA (ssDNA)–binding protein in human cells (Prakash and Borgstahl 2012). It is a trimeric protein complex with multiple DNA-binding domains, mainly in its first subunit, and is heavily involved in DNA replication, repair, and recombination (Oakley and Patrick 2010; Prakash and Borgstahl 2012; Qureshi et al. 2012). Similar to POT1, RPA acts to unfold telomeric G4Qs. Unlike POT1, it approaches from the 5′-3′ direction and is also able to unfold both parallel and antiparallel G4Qs (Ray et al. 2014). Crystal structures exist for the ssDNA complex, but not yet with the G4Q (Bochkarev et al. 1997; Bochkareva et al. 2001, 2002).

### Human CST (CTC1–STN1–TEN1)

Human CST is a trimeric complex that plays a number of roles that aid in replication, largely at the replication fork, but also has critical actions at the telomeres, ensuring their stability (Bhattacharjee et al. 2017). CST binds telomeric G4Qs, unwinds them, and facilitates the production of the complementary C-rich strand in a process known as C-strand fill-in (Bhattacharjee et al. 2017). More specifically, the CTC1 is the subunit containing the DNA-binding domain, whereby the TAGG of the repeats is found to be tightly associated with the protein in its unwound form, per cryo-EM studies (Lim et al. 2020).

### Breast cancer type 1 susceptibility protein (BRCA1)

BRCA1 is a tumor suppressor protein, whereby functional dysregulation from mutations confer a high risk of breast and ovarian cancers (Ford et al. 1994). The exact mechanism of tumor suppression is not fully known, but is heavily involved in DNA repair, cell cycle regulation, and, more recently, regulation of telomeres (Ballal et al. 2009). The latter function has since been supplemented with in vitro data showing binding to G4Qs (Brázda et al. 2016). Thus, there is also a good case for BRCA1 as a direct G4BP; however, further structural data describing this interaction is needed.

### Heterogeneous nuclear ribonucleoprotein A1 (hnRNP A1) and Unwinding Protein 1 (UP1)

hnRNP A1 is a ribonucleoprotein involved in RNA transport, alternative splicing, microRNA biogenesis, and transcriptional control (Hudson et al. 2014). The protein has two nucleic acid–binding sites capable of interacting with RNA or DNA. UP1 is the proteolytic cleavage product of hnRNP A1 that retains those two binding domains. These two proteins are able to unfold G4Qs to facilitate telomerase binding at the telomeres to enable telomere lengthening (Hudson et al. 2014). With focus on the UP1, mutational studies on the hexameric repeat region shows a consensus binding sequence of d(nYAGn), where Y is either a thymine or cytosine residue (Ding et al. 1999; Myers et al. 2003). Crystal structures have been solved of UP1 bound to RNA (Morgan et al. 2015) and ssDNA (Ding et al. 1999), both highlighting the dimerization of the UP1 molecules creating linear channels for the nucleic acids in an antiparallel fashion. How the protein initially binds the telomeric G4Q is not yet clear, but in vitro studies show the UP1 is able to degrade G4Qs and its affinity for single stranded linear nucleic acid far outweighs the structured form, likely adding an energy-favorable driver to its unfolding.

The hnRNP is also able to bind and unfold G4Qs in promoter regions, including the KRAS promoter G4Q and the TRA2β promoter. The KRAS gene is central to cell-growth signaling pathways, and the presence of the G4Q in its promoter acts as a repressor (Xodo et al. 2008). The hnRNP binding and unfolding enable the transcription to resume, which in the case of some cancers, like pancreatic adenocarcinoma, has detrimental effects (Xodo et al. 2008). Interestingly, researchers have developed a potential therapeutic G4Q decoys to bind this and other G4BPs that may act on this G4Q (Cogoi et al. 2009; Podbevšek and Plavec 2016). Another G4Q promoter target of hnRNP A1 is the TRA2β4 exon 2, regulating alternative splicing of exon 2, whereby G4Q binding and subsequent unfolding facilitates inclusion of the exon 2 (Nishikawa et al. 2019). Binding and functional studies are available on these, but there are no detailed interaction studies as of yet.

Regarding hnRNP’s role in binding RNA G4Qs, hnRNP A1 was shown to be involved in mRNA transport, chaperoning the RNA into the cytoplasm and present near translation (von Hacht et al. 2014). This information was determined from pull-down studies using the matrix metalloproteinase MT3-MMP (also known as MMP16) and the actin-related protein 2 (ARPC2) and confirmed with functional and mutational studies (Serikawa et al. 2018; von Hacht et al. 2014).

### Bloom protein (BLM)

BLM is a highly conserved RecQ-like helicase that has functions in DNA repair and telomeric stability. The helicase acts in a 3′-5′ direction, functioning on the leading strand of replication (Sauer and Paeschke 2017). Unfolding is mediated by two domains, the RecQ C-terminal domain (RQC) and HRDC, which bind and unfold in sequential steps. The process is not necessarily direct, and there can be refolding and unfolding transitions throughout the process (Chatterjee et al. 2014). Unfolding of the G4Q appears to be made easier with a longer trailing segment, at least 6–8-nt long on the 3′ end, likely implying binding, or alternatively favorable positioning of the BLM helicase (Budhathoki et al. 2014). It has also been shown that unlike many other helicases, the BLM does not appear to need adenosine triphosphate (ATP) to facilitate its unfolding (Budhathoki et al. 2014).

### Werner’s syndrome protein (WRN)

WRN is another RecQ-like helicase and a counterpart of BLM. It exerts its helicase function on the lagging strand of replication (Sauer and Paeschke 2017). Binding and unwinding are similarly facilitated by the RQC and HDRC domains, which are conserved throughout this family of helicases (Chatterjee et al. 2014; Lerner and Sale 2019).

### Preimplantation factor-1 (Pif1)

Pif1 is a highly conserved helicase across many domains of life and has roles in maintaining genome stability (Paeschke et al. 2013; Zhou et al. 2014). It acts to unwind G4Qs, including telomeric G4Qs, moving in a 5′-3′ direction. There has been some conflicting evidence in the literature regarding the nature of this enzyme’s helicase functioning, including its ability to rapidly unfold the G4Q but not the dsDNA beyond or that unwinding is slow, but that it does both (Byrd and Raney 2015; Hou et al. 2015; Zhou et al. 2014). A fairly recent study highlights the importance of experimental conditions and shows that while the enzyme is ATP-dependent, some G4Q unwinding can occur in the absence of ATP, perhaps due to transient folding and unfolding of a less stable G4Q (Byrd et al. 2018). This allows the Pif1 to bind the ssDNA can be trapped by the helicase, creating a longer ssDNA overhang that allows more Pif1 enzymes to bind. After a multistep G4Q unwinding, Pif1 is able to proceed with downstream dsDNA unwinding, the rate of which is dependent on the rate of the G4Q unfolding (Byrd et al. 2018; Zhou et al. 2014). There are crystal structures available for Pif1 bound to short DNA oligomers and to dsDNA (Su et al. 2019), but none yet incorporating a G4Q.

## G4BPs targeting promoters

Gene promoter regions are another site of G4Q formation and subsequent site of G4Q-binding protein recruitment. The most well-studied are those preceding oncogenes, in particular the c-MYC promoter. The c-MYC promoter and related oncogene is considered one of the master regulators in cancer biogenesis where overexpression upregulates many aspects of cellular growth and metabolism (Chen et al. 2018a; Dang 2012; Miller et al. 2012). Unsurprisingly, there are a number of proteins that bind and regulate this promoter, described in various reviews (Dang 2012; Miller et al. 2012; Wang et al. 2021). Those known specifically to bind G4Qs will be highlighted here, recognizing that this list may not be exhaustive. In addition to the c-MYC promoter, many other promoters containing G4Qs have been identified, some acted upon by the same G4BPs highlighted above. Evidence for the functional roles of G4Qs in these sites is evolving alongside the structural data, as our understanding increases regarding their roles in human diseases. The G4BPs described here are the DEAH box protein 36 (DHX36), non-metastatic factor (NM23-H2), nucleolin, MYC-associated zinc finger (MAZ), Specificity protein 1 (Sp1), Yin Yang 1 (YY1), poly (ADP-ribose) polymerase 1 (PARP1), and the transcriptional helicases, and xeroderma pigmentosum type B and D (XPB and XPD).

### D-E-A-H box protein 36 (DHX36; also known as G4 resolvase 1 (G4R1), MLE-like protein 1 (MLEL1), and RNA helicase associated with AU-rich elements (RHAU))

DHX36 is a helicase belonging to the DEAH box family of enzymes and has been shown to bind and unwind both DNA- and RNA-G4Qs (Creacy et al. 2008; Lattmann et al. 2010). Its most well-characterized roles relating to G4Qs are in resolving the G4Qs in RNA, hence one of its aliases; however, there are also studies describing their role in DNA including in the telomeres (Booy et al. 2012; Lattmann et al. 2011; Sexton and Collins 2011) and in promoter regions (Huang et al. 2012).

**Fig. 3:**
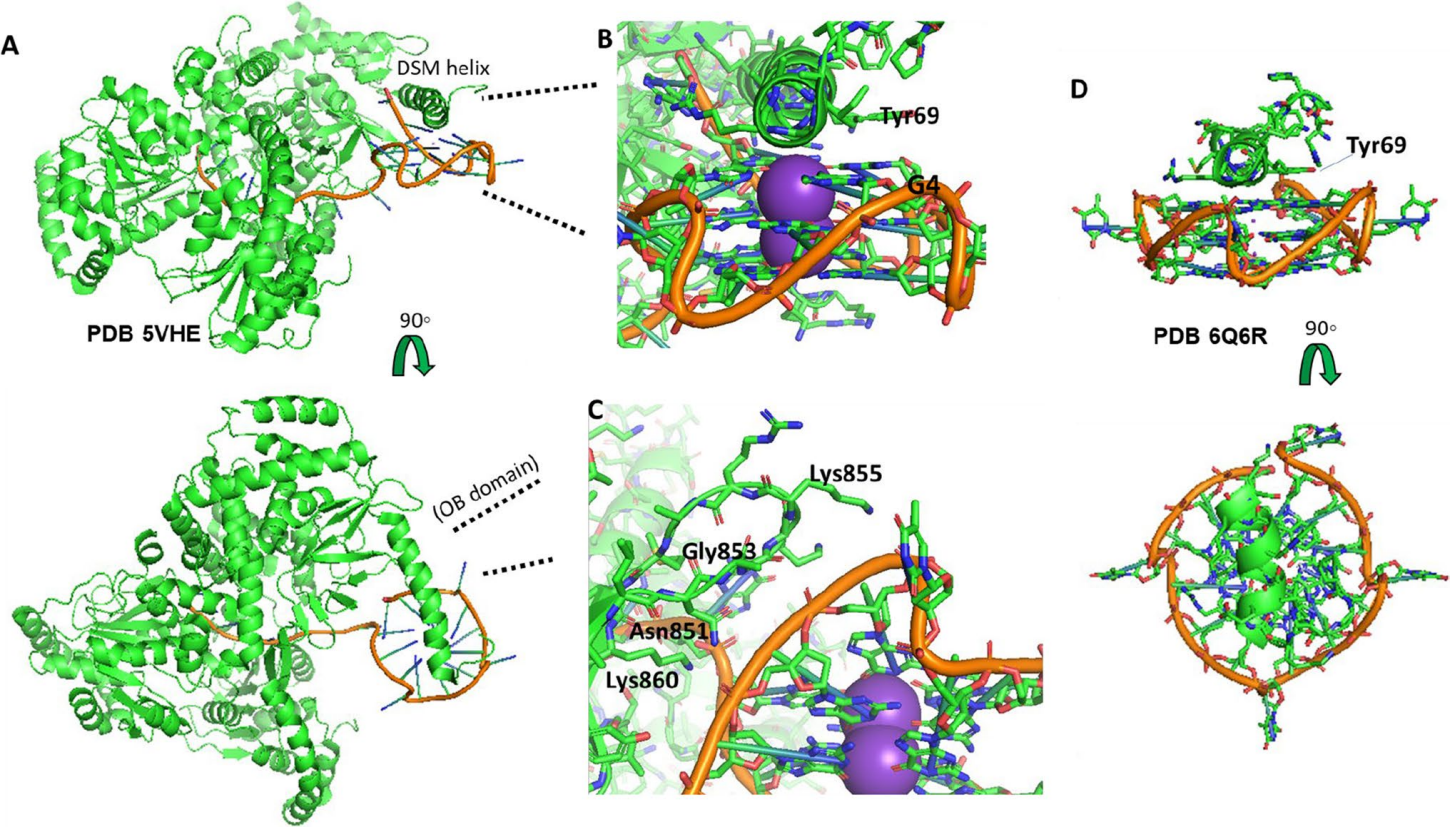
Example of top-stacking—DHX36 with c-MYC promoter region G4Q. **A** High-level orientation of the structural arrangement showing the DSM helix sitting atop the G4Q, the lateral OB domain loop contacting the G4Q from the side, while the G4Q is pulled through the RecA-like domains (see text; PDB 5VHE); **B** DSM helix showing the Tyr69 oriented parallel with an upper guanosine from the tetrad facilitating π-π stacking. Other hydrophobic residues make up the remainder of the downward facing helical residues (Ile65, Trp68, and Ala70); **C** OB domain showing the proximity for the extensive hydrogen-bonding network between the phosphate backbone of the G4Q and Lys860, Asn851, Gly853, and Lys 855. **D** Independent study of the DHX36 DSM domain with c-MYC showing similar top-stacking binding mode (PDB 6Q6R)

A co-crystal structure of bovine DHX36 with the c-MYC promoter region G4Q provides insight into this interaction, supporting prior structural data and revealing new information about its binding mode and action (Fig. 3A; PDB 5VHE) (Chen et al. 2018b). First, the helicase is comprised of two RecA-like domains that together create a small positively charged channel big enough to accommodate a single strand of DNA. A further C-terminal domain consists of the oligosaccharide-binding domain (OB) and the G4Q-binding helix, or DHX36-specific motif (DSM). The DSM helix sits atop the G4Q’s 5′ face, while the OB domain contacts both the G4Q side and trailing single-stranded 3′-terminal DNA. Independent crystallography and NMR studies show that the quadruplex forms a stable, 3-tetrad structure in solution (Fig. 3D) (Ambrus et al. 2005; Heddi et al. 2020; Phan et al. 2004). In the co-crystal structure, however, the 3′-most guanosine trio has been “tugged” downward, resulting in the upper tetrad being formed with neighboring thymine and adenosine as the DNA strand is pulled into the cavity—a description and schematic best viewed in the original paper (Chen et al. 2018b). This allows one to envision the further collapse of the G4Q as more of the nucleotides are pulled through, linearizing the structure.

Focusing on the DSM helix and G4Q interaction in more detail (Fig. 3B), the G4Q-facing residues of the DSM create a hydrophobic surface with its Ile65, Trp68, Tyr69, and Ala70, enabling stacking with the bases of the upper G4Q tetrad. This binding mode is akin to many small-molecule G4Q binders that have been studied (Collie et al. 2012; Medeiros-Silva et al. 2017; Nielsen et al. 2014; Ohnmacht et al. 2015; Read et al. 2001). An arginine (Arg63) of the helix reaches over to bind one of the phosphate-backbone oxygen. Studies looking at the dissociation constants, *K*_*d*_, of full-length versus truncated DHX36 in combination with the G4Q report *K*_*d*_ values of < 10 pM and 310 nM, respectively (Booy et al. 2012; Giri et al. 2011; Lattmann et al. 2010). This suggests that at the very least the DSM alone is not the sole contributor to the binding. Indeed, as the co-crystal structure highlights, the OB domain is also involved in this interaction. It contains two loops—one interacts with the sugar phosphate backbone of the G4Q, forming extensive hydrogen bonds (largely with residues Lys860, Asn851, Gly853 and Lys 855; Fig. 3C), and the second loop interacts with the trailing 3′-strand (Chen et al. 2018b). One question that arises is whether the helicase “processes” all single-stranded DNA in this manner or whether it uses the G4Q as a recognition signal to then attach to the strand and unwind it. Certainly, the *K*_*d*_ of the interaction where a G4Q that is being pulled through a full-length DHX36 helicase will likely be much tighter than that of the helix and G4Q in solution, but it does not give us the initial binding drive, but rather an averaged of what is likely a favorable overall action.

In another study, DHX36 is shown to interact with the Yin Yang 1 (YY1) gene, a gene that encodes for a multifunctional protein involved in tumorigenesis (Gordon et al. 2006). A G4Q exists in both the promoter of the YY1 gene and in the 5′-UTR of the mRNA it produces (Huang et al. 2012). Interestingly in their study, DHX36 was found to bind only to the DNA G4Q and not the RNA, despite both being parallel quadruplexes. This could imply a selectivity that is based on the deoxyribose chain. An alternative interpretation could be the need for trailing 3′-tail to facilitate binding of which was not included in the comparator RNA in this study (Huang et al. 2012). Other studies have also shown DHX36 to selectively bind parallel DNA G4Qs (Heddi et al. 2020; Tippana et al. 2019, 2016), but also that the single-stranded tail plays an important role in that binding (Yangyuoru et al. 2018). This, coupled with the co-crystal structure above showing OB domain binding to the ssDNA, further supports this (Fig. 3C).

A more recent and important study involving DHX36 and synthetic G4Qs nicely employs small-molecule Förster resonance energy transfer (smFRET) and computational modeling to describe the process of G4Q unwinding in both DNA and RNA, which has distinct differences (Tippana et al. 2019). Again, while the DHX36 binding to G4Q DNA facilitates unfolding independent of ATP, in a presumed “tugging” and G4Q rearrangement, DHX36 binding to RNA G4Qs enable a stably unfolded state, which is followed by repetitive cycling of refolding in an ATP-dependent manner. The work was also complemented by mutational studies that show rapid dissociation of the complex with ATP hydrolysis when the DSM helix was mutated. This was in contrast to mutations in the sites responsible for binding the 3′-trailing strand (the RecA2 and OB domains), which resulted in erratic DHX36 motions that was unable to facilitate the G4Q refolding, but that maintained binding (Tippana et al. 2019). Another interesting distinction the authors also note with one of their mutations, Y69A in the DSM helix, is that the DNA G4Q readily washed off in one of its processing steps, while the same mutant RNA G4Q held fast (Tippana et al. 2019). This could imply additional stabilizing bonds with the hydroxyl groups in backbone of the RNA, or at the very least, an altered binding mode.

### Non-metastatic factor (NM23-H2)

Non-metastatic factor (NM23-H2; nucleoside diphosphate kinase) is a transcriptional factor that binds the nuclease hypersensitive element (NHE) in the c-MYC promoter that acts as a transcriptional silencer (Sengupta et al. 2019). Using chromatin immunoprecipitation, reporter assays, and FRET studies, NM23-H2 has been shown to bind and resolve this G4Q enabling transcription to proceed (Thakur et al. 2009). Importantly, NM23-H2 is also one of several G4BPs that contains an arginine-glycine-glycine (RGG) motif that is presumed to play a role in G4Q recognition and/or binding (see “Commonalities amongst G4BPs linked to binding modes”).

### Nucleolin

Nucleolin is a multifunctional phosphoprotein found throughout the cell, but in high abundance in the nucleolus, for which it is named. Its many roles include ribosomal biogenesis, chromatin remodeling, transcription, and apoptosis (Ginisty et al. 1999; Mongelard and Bouvet 2007; Tajrishi et al. 2011). It is also well-known to bind G4Qs, including the c-MYC promoter G4Q, where binding, unlike with NM23-H2, stabilizes the G4Q leading to reduced transcription (González et al. 2009). Nucleolin binding has been shown in vitro with the G4Qs of the bcl-2, hTERT, VEGF, RET, PDGF-A, and c-kit promoters but the functional significance of these interactions has not yet been elucidated (González et al. 2009; Lago et al. 2017). It has also been shown to bind the hexanucleotide repeat expansion (HRE) of the *C9orf72* gene that is linked to the neurodegenerative diseases, amyotrophic lateral sclerosis (ALS), and frontotemporal dementia (FTD) (Haeusler et al. 2014). Where normally the nucleolin binds these repeats (typically 2–8 repeats of GGGGCC), in those affected, repeats can number in the thousands resulting in nucleolar dysfunction (Haeusler et al. 2014).

Regarding the nature of its binding, nucleolin has been shown to have a preference for parallel G4Qs over anti-parallel ones (González et al. 2009). Further insights into its binding with the c-MYC G4Q are gained from the studies looking at loop length mutational analysis (González et al. 2009; Lago et al. 2017). Here, a series of G4Qs with varying loop lengths reveal that while the shorter loop lengths create a more stable G4Q, nucleolin prefers to bind G4Qs with somewhat lower stability, and in particular those with at least one long loop (3–7 nt) (Lago et al. 2017).

Nucleolin, like NM23-H2, is also an RGG-motif-containing G4BP. Nucleolin was actually the first protein to have this identified whereby a repeating RGG motif (9 repeats) is found in the protein’s C-terminal region (Hanakahi et al. 1999). There is also an additional RNA recognition motif (RRM) upstream of this repeating unit that was known to bind single-stranded DNA, but also more recently shown to independently bind G4Q DNA (Hanakahi et al. 1997, 1999). Together, the two regions within nucleolin are hypothesized to be able to help fold and stabilize the G4Q. The existence of this common RGG motif in other enzymes known to bind G4Qs, namely, hnRNP A1 (Ding et al. 1999) and NM23-H2 (Dexheimer et al. 2009), has laid highlight to these motifs as recognition domains; however, the specifics of their interaction has yet to be fully elucidated (see “Commonalities amongst G4BPs linked to binding modes”).

### MYC-associated zinc finger (MAZ)

MAZ is a six zinc finger protein essential to transcription and chromosomal organization (Xiao et al. 2021). For each of these functions, MAZ binds G-rich, G4Q forming regions. In the C-MYC promoter, a G4Q represses transcription and can be unwound by the MAZ protein to derepress it (Siddiqui-Jain et al. 2002a). Similarly, in the HRAS promoter, there are two upstream quadruplexes that maintain transcriptional repression, which when bound and unwound by MAZ are able to proceed with their downstream processing (Cogoi et al. 2014; Membrino et al. 2011). MAZ can also bind and unwind the Pur-1 G4Q of the IDDM2 locus to enable transcription (Lew et al. 2000). Interestingly, this G4Q-unwinding action of MAZ is unique thus far to this zinc finger protein, as other zinc finger proteins do not yet claim this action against G4Qs.

Furthermore, MAZ has also been shown to bind the upstream G4Q of the KRAS promoter enabling transcription. This insight has led to unique approach of developing G4Q-decoys for the zinc finger in pancreatic cancer cells (Cogoi et al. 2010, 2013). Despite its many roles, a structural representation of MAZ with one of its G4Qs has yet to be documented.

### Specificity protein 1 (Sp1)

Specificity protein 1 (Sp1) is a ubiquitous transcription factor and member of the Krüppel-like family, which are a group of transcriptional regulators containing a triple zinc finger motif (Black et al. 2001; Vizcaino et al. 2015). Binding sites are variable including GC-boxes and G-rich quadruplex forming regions (Raiber et al. 2012). In vitro evidence exists suggesting that all three of the zinc fingers contribute to the DNA interaction, which has been a notable feature of zinc finger proteins that has also been useful in the design of synthetic zinc fingers (Al-Naama et al. 2020; Eom et al. 2016; Jamieson et al. 2003; Razin et al. 2012; Yokono et al. 1998). These manipulable features are for its interaction with *linear* DNA, however, as little structural data exists with Sp1 in combination with the G4Q form as of yet. It is beyond the scope of this review, but it may be interesting to review the details of prior such zinc finger interaction studies to determine whether the experimental conditions of each would have supported the formation of G4Qs and thus have the potential to infer results on these structures. Of the promoter regions Sp1 is known to interact and regulate, those containing G4Qs at the site of Sp1 binding are c-KIT (Da Ros et al. 2021), HRAS (Cogoi et al. 2014; Membrino et al. 2011), and VEGF (Yokono et al. 1998). In the case of HRAS, Sp1 binding to its G4Q promoter acts as a transcriptional repressor and seems to also require MAZ (another G4Q-binding zinc finger protein, noted above) for its action (Membrino et al. 2011).

### Yin Yang 1 (YY1)

Yin Yang 1 is yet another zinc finger protein recently found to target G4Qs (Li et al. 2021). Interestingly, it itself is under the control of a G4Q (see section on DHX36) (Huang et al. 2012). This zinc finger protein has integral roles in transcription, acting as a regulator of promoter-enhancer loops (Gordon et al. 2006; Li et al. 2021; Weintraub et al. 2017). A recent study nicely employs a mix of proximity ligation assays and chromatin immunoprecipitation assays to show how YY1 can bind two sites of DNA G4Qs and through dimerization bring the two pieces of DNA together, supporting its role in the positioning of promoter and enhancer sites (Li et al. 2021). Regarding its mode of binding, it was noted that using the G4Q stabilizers, TMPyP4 and pyridostatin, G4Q-binding and promoter-enhancer loop formation was greatly reduced, supporting the likely possibility that there are at least some shared points of contact in their respective interactions (Li et al. 2021).

### Poly (ADP-ribose) polymerase 1 (PARP1)

PARP1 is a ubiquitous nuclear zinc protein that has a high affinity for damaged DNA and has key roles in chromatin remodeling and gene expression. It has been shown in vitro to bind the G4Qs in the promoter regions of oncogenes c-KIT, c-MYC, and KRAS (Cogoi et al. 2010; Edwards et al. 2020; Fekete et al. 2012; Soldatenkov et al. 2008). Functionally, binding at c-KIT results in catalytic activation of PARP1 (Soldatenkov et al. 2008). Evidence of gene activation upon PARP1 binding also exists for the G4Q upstream from KRAS (Cogoi et al. 2010). Furthermore, studies examining PARP1’s specificity for certain G4Qs revealed the preference for parallel G4Qs, as evidenced by lack of binding to the hybrid G4Q-forming hTEL promoter region (Edwards et al. 2020). The same group considered the loop specificity as part of the incorporation, revealing that while a shorter loop enabled the G4Q bind with greater affinity to PARP1, the pentanucleotide loop in its original form (CGAGC) was required for activation of PARP1, indicating a role and incorporation of this lateral G4Q loop into the overall functional binding epitope (Edwards et al. 2020). There are X-ray- and NMR-based models of the PARP1 protein in combination with helical DNA and strand breaks (Ali et al. 2012; Bilokapic et al. 2020; Eustermann et al. 2015; Langelier et al. 2011, 2012; Patel et al. 2014), but none yet exists of PARP1 in complex with a quadruplex. Speculation would lead one to believe the same region would be responsible, but how the protein conforms to do so is still unknown.

### Transcriptional helicases xeroderma pigmentosum type B and D (XPB and XPD)

XPB and XPD are both helicase components of the transcription factor II human (TFIIH) complex, an eleven-subunit transcriptional complex that plays a central role in transcription and nucleotide excision repair (NER) (Drapkin et al. 1994; Gray et al. 2014). The XPD helicase has been shown to unwind G4Qs in a 5′-3′ direction in an ATP-dependent manner; the XPB helicase acts in a 3′-5′ direction has been shown only to bind, but not unwind the G4Q (Gray et al. 2014). While there is a crystal structure available of the XPD Arch domain with the protein MAT1 (Peissert et al. 2020), there is no complex with DNA.

## G4BPs involved in DNA replication

### Fanconi Anemia Complementation group J (FANCJ, also known as BRIP1 or BACH1)

FANCJ belongs to the XPD-like group of helicases and facilitates a number of processes including DNA replication, homologous recombination (HR), and interstrand DNA crosslink (ICL) repair (Wu and Spies 2016). Its actions on DNA replication are facilitated through the binding and unwinding of G4Q DNA in the 5′-3′ direction. The helicase appears to be critical to the process, such that in its absence, DNA replication persistently stalls at the G4Q site (Castillo Bosch et al. 2014). FANCJ has been shown to unwind both intramolecular and intermolecular G-quadruplexes in vitro (Bharti et al. 2013). While it is unclear how broad the scope of G4Q binding may be for this helicase, binding studies have been performed using the telomeric G4Q ((TTAGGG)_n_) and a panel of uni-, bi-, and tetramolecular G4Qs (Bharti et al. 2013; Lowran et al. 2020). The key residues of FANCJ involved in the interaction are the alanine-alanine-lysine-glutamine (AAKQ) motif. Furthermore, recent results suggest the AAKQ interaction may be with the TTA loop region of the G4Q (Lowran et al. 2020).

There is a homologous G4BP found in *Caenorhabditis elegans* known as Dog-1 that supports the FANCJ helicase results, but also noted here because it was also one of the first helicases shown to interact with G4Qs in vivo (Cheung et al. 2002; Kruisselbrink et al. 2008). It similarly has critical roles in DNA repair, namely, ICL, and like FANCJ, its actions on G4Qs are in the 5′-3′ direction. From the above-noted mutational studies, there also does not appear to be a specific sequence or G4Q form this enzyme preferentially binds, fitting with a non-specific DNA G4Q helicase.

## G4BPs targeting RNA

RNA G4Qs, like their DNA counterparts, also play roles in critical cellular processes, including termination of transcription, telomerase activity, alternative splicing, and regulation of translation (Kharel et al. 2020; Lyu et al. 2021; Song et al. 2016). Structurally, RNA G4Qs are similar to those of DNA, but are much more thermodynamically stable, owing to the more extensive network of hydrogen bonding afforded from the ribose C2′ hydroxyl groups (Arora and Maiti 2009; Joachimi et al. 2009; Zhang et al. 2011, 2010)(noted above). Another feature of naturally occurring RNA G4Qs is that they are essentially all parallel G4Qs (with few exceptions (Xiao et al. 2017)). That being said, the structural similarities between RNA and DNA G4Qs still hold, it is not surprising to find that many of the proteins able to bind DNA G4Qs are also able to bind RNA G4Qs. Of those G4BPs already described above, those with identified binding and/or roles in both include one of the most well-studied RNA G4BPs, DHX36, and nucleolin and the hnRNPs. Other RNA G4BPs include DHX9, the heterogeneous nuclear ribonucleoproteins (hnRNP), serine/arginine-rich splicing factors (SRSF), the AF4(ALL1-fused gene from chromosome 4)/FMR2(fragile X mental retardation 2) (AFF) family of proteins and, of course, ribosomal proteins.

### D-E-A-H box protein 9 (DHX9; also known as nuclear DNA helicase II (NDH II) and RNA helicase A (RHA))

DHX9 is an ATP-dependent helicase, similar to DHX36 described above, belonging to the SF2 superfamily of helicases (Chakraborty and Grosse 2011). It also has the ability to unwind G4Qs, with a preference for RNA substrates (Murat et al. 2018). A transcriptome-wide analysis reveals the importance of DHX9 (and DHX36) in the translational control of many transcripts, whereby the G4Qs will cause ribosomal queuing and potential alternate translations if not unwound (Murat et al. 2018). While DHX36 has the DSM helix providing the top-stacking interaction, DHX9 does not have a similar motif. It does contain an RGG box motif in its C-terminus, however, which may be able to facilitate G4Q binding as has been seen in several other G4BPs (Chakraborty and Grosse 2011). DHX36 also has an RGG-box region in its N-terminus, but this region does not appear to have interaction with the G4Q (Fig. 2), though the N-terminal region is often truncated in many studies on the enzyme (Chen et al. 2018b).

### Serine/arginine-rich splicing factors (SRSF)

SRSFs are a highly conserved family of RNA binding proteins involved in alternative and constitutive splicing, mRNA transport, translation, mRNA decay, and genome stabilization (Long and Caceres 2008). They contain an N-terminal RNA recognition motif (RRM) and C-terminal arginine-serine (RS)-domains that appears to have a strong regulatory role in the proteins’ functioning, whereby phosphorylation of these domains can enhance binding to other RS-containing splicing proteins bringing them together (Lin and Fu 2007; Long and Caceres 2008). SRSF was originally identified to bind G4Q RNA from pull-down studies (noted above in hnRNP section) using MMP16 and ARPC2 (Serikawa et al. 2018; von Hacht et al. 2014). Further structural information is not yet available, nor whether G4Q-binding plays a specific role in the functional aspects of these groups of proteins.

### Fragile X Mental Retardation Protein (FMRP)

**Fig. 4:**
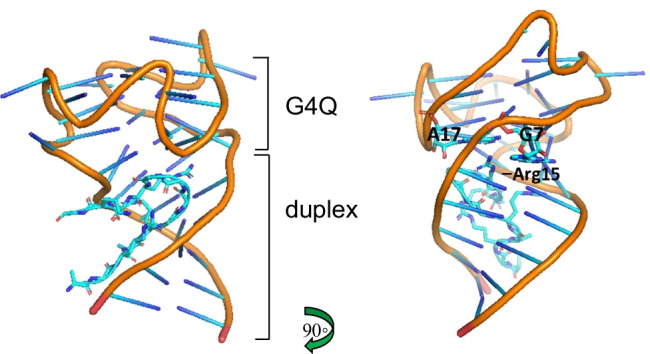
FMRP’s 13-amino acid β turn folding into the groove at the junction of duplex and G4Q DNA (PDB 5DE5). The uppermost amino acid, Arg15, interacts with G7 and A17 nucleotides, which are not part of the G4Q structure. Binding is thought to promote stabilization of the G4Q (see text)

Fragile X Mental Retardation Protein (FMRP) is an RNA regulatory protein belonging to the AF4 (ALL1-fused gene from chromosome 4)/FMR2(fragile X mental retardation 2) (AFF) family of proteins that chaperones mRNA from the nucleus and has roles in transcriptional control. Its malfunction is linked to several human disorders, including Fragile X syndrome and autism (Darnell et al. 2001; Phan et al. 2011). FMRP binds G4Qs via its RGG motif and this alone appears to be necessary, at least for binding to sequence clone 1 (*sc1)* RNA, per extensive mutational studies [*sc1* was selected from a pool of random RNA oligomers (*sc*’s) to bind FMRP] (Darnell et al. 2001). Both crystal structure and NMR data are available on this motif’s interaction with the G4Q in *sc1*, which shows the RGG motif forming a β-turn with 13 of the peptide’s amino acids and fitting nicely into the junction at the base of the G4Q and the duplex RNA (Fig. 4—PDB 5DE5) (Phan et al. 2011; Vasilyev et al. 2015). In this structure, the RGG motif bound to two consecutive G-C base pairs of the duplex, with its Arg8, Arg10 and Gly11 and the type I β-turn (with intramolecular hydrogen bond between Gly12 and Arg15) extends upward towards the G4Q. Its furthest most reaching residue, Arg15 however, only interacts with the G7 and A17 nucleotides, neither of which are part of the specific G4Q structure (Fig. 4). Just the same, it is speculated that the stabilization afforded through this loop binding in the junction between the duplex and G4Q may be a driver of stability (Vasilyev et al. 2015).

### Ribosomal proteins

G4Qs are found in the 5′-UTR of many mRNAs. From the MMP16 and ARPC2 pulldown studies described above, ribosomal proteins were identified from both the 60S and 40S subunits (von Hacht et al. 2014). G4Qs in the 5′-UTRs are thought to act as more of a translational regulator, slowing down protein translation while the ribosome waits for a helicase to unwind the structure (Bugaut and Balasubramanian 2012; Huppert et al. 2008). It may be possible that the recognition of the G4Qs by ribosomal proteins plays further roles, but this has yet to be determined.

## G4BPs that degrade G4 DNA

Research on G4BPs with the ability to degrade G4 DNA remains an evolving field, however, the two of which we have the most information are the human nuclease, GQN1 (G quartet nuclease 1) and the *Saccharomyces cerevisiae* Mre11 protein (ScMre11p) (Ghosal and Muniyappa 2005, 2007; Sun et al. 2001). These proteins are nucleases that appear to use the G4 as an anchor for cleavage of the G4 DNA at a site nearby, degrading the single strand.

GQN1 cuts the single-stranded DNA region 2–5 nt 5′ of the barrel formed by stacked G-quartets and is independent of the upstream sequence. The nuclease does not degrade duplex or single-stranded DNA or G4 RNA (Sun et al. 2001). Another nuclease is the *Saccharomyces cerevisiae* Mre11 protein (ScMre11p), which also shows high binding affinity for G4 DNA over single- or double-stranded DNA. In this case, the binding of ScMre11p to G4 DNA facilitates endonucleolytic cleavage at G residues that flank the G-quartets (Ghosal and Muniyappa 2005, 2007).

There are currently no enzymes known to be able to degrade the G4Q structure in its wound form.

## Commonalities amongst G4BPs linked to binding modes

The different functions G4BPs have are likely dictated or a least heavily linked to the binding mode the protein assumes in interacting with a G4Q. G4BPs that unwind a multitude of different G4Qs, such as DHX36, cannot be too selective toward G4Qs. Thus, a top-stacking binding mode would appear to be more practical—stabilizing the stacked column of G4-tetrads while another part of protein unwinds the G4Q like a spool (Fig. 3).

A subset of G4BPs are the groove-binders, such as nucleolin or FMRP, whereby the presence of an RGG motif, shown to fold into a β-loop, is able to fit into a grove of the G4Q and act as a stabilizer (Masuzawa and Oyoshi 2020; Vasilyev et al. 2015). The presence of the RGG motif alone is not enough to define its function, however, as other G4BPs contain this region (i.e., hnRNP A1 and NM23-H2) and these proteins have differing roles from stabilizing to unwinding (Dexheimer et al. 2009; Ding et al. 1999; Huang et al. 2018; Thandapani et al. 2013). From the stabilization perspective, there at least appears to be some support in the FMRP data (Fig. 4; Phan et al. 2011; Vasilyev et al. 2015)). Given the abundant links with the repeating RGG-motif in many highly regulatory proteins, there is much still to explore with how this contributes to the interaction and functioning around the G4Q.

### A comment on zinc finger protein binding

**Fig. 5:**
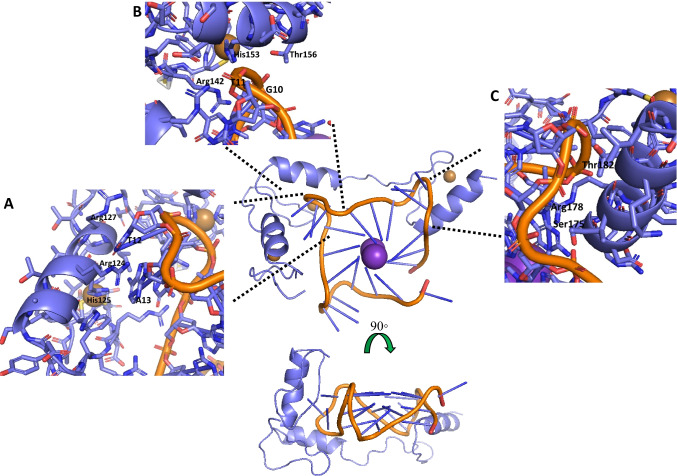
Example of loop-binding mode—synthetic zinc finger, Gq1 targeting a telomeric G4Q. Computationally derived model (PDB from Ladame, et al. 2006), showing overall arrangement (center) and key residues from **A** the first “finger,” whereby His125, Arg124, and Arg127 interact with the two outward-directed T12 and A13 nucleotides; **B** the second “finger,” where His153 and Thr156 bind with phosphate backbone of G10, while Arg142 wraps under to bind the other protruding nucleotide, T11; and **C** the third “finger,” where the Ser175, Arg178, and Thr182 create extensive H-bonds with the phosphate backbone of the loop

Zinc finger proteins (ZFP) are an interesting subset of G4BPs that warrant highlighting separately. There are currently no experimentally derived high-resolution structures of a ZFP bound to a G4Q. There is one very nice combination biophysics-computational modelling study of a synthetic ZFP, Gq1, a zinc finger protein originally derived from a phage display library coupled with a systematic series of biophysical binding assays (Isalan et al. 2001; Ladame et al. 2006; Patel et al. 2004). In the study, researchers compared binding of Gq1 and the yeast zinc finger homologue, Zif268 to telomeric G4Q or Zif268’s (duplex) dsDNA binding sequence. In particular, they systematically swapped out the “fingers” of one for the other to determine which had the greatest effects on G4Q affinity and selectivity. Their results showed that any one finger of Gq1 could be replaced with the corresponding finger of Zif268, without losing quadruplex affinity or duplex discrimination, but when two fingers were exchanged, where one was the second finger, both tenfold reductions in binding and loss of G4Q-duplex discrimination resulted (Ladame et al. 2006). The modelling studies go on to support the biophysical data, providing a resultant lowest energy coordinate file, whose arrangement is outlined in Fig. 5. Here, the model supports more of the loop-binding mode of interaction. The first of the “fingers” binds with two of the three protruding nucleotides (T12 and A13), while the second “finger” binds the third loop nucleotide (T11) and simultaneously holds the phosphate backbone (Fig. 5A, B). The third “finger” focusses on the neighboring loop, extensively binding the phosphate backbone of the outpouching loop (Fig. 5C).

This work provides us with some of the first supporting data for how this class of proteins may in fact interact with these structures.

## G4Q loops affording the basis for selectivity?

With its many critical cellular roles, it would follow logically that G4Qs must have some degree of selectivity to bind the correct G4BPs. This concept has been demonstrated in many studies of G4Q-G4BP interactions (Heddi et al. 2020; Huang et al. 2012; Lago et al. 2017). With the barrel core is similar amongst G4Qs, it is the orientation and length of the loops that make one G4Q different from the next and likely the mechanism by which selectivity is afforded.

G4Q stability is inversely linked to loop length—the shorter the loop, the more stable the G4Q. Guedin et al. performed a nice systematic assessment of this whereby they studied the melting temperatures (*Tm*) of a large panel of G4Q with varying loop lengths, showing that a length greater than 9 nt had a destabilizing effect on the structure, especially if multiple longer length loops were incorporated (Guédin et al. 2010). The c-MYC promoter G4Q is an example of a highly stable, short loop-length G4Q, while the TRA2β has a single 8-nt loop (Table 1). It is possible that only some types of helicases are able to overcome a more stably-folded G4Q and be identified as a binding protein, such as that of the DHX36 helicase and its action on the c-MYC, whereby crystal structure evidence suggests it pulls the G4Q through its RecA-like channel (Fig. 3A; PDB 5VHE) (Chen et al. 2018b).

**Table 1:** G4-quadruplex binding proteins (G4BPs)

	G4-sequence	Function, if known	Comments
Telomeric G4BPs			
POT1	(TTAGGG)n	Unfolding of G4 (and refolding with POT1-TPP1 complex	Selective to anti-parallel G4Qs
RPA	(TTAGGG)n	Unfolds G4Qs	Unfolds both parallel and anti-parallel G4Qs
Human CST	TTAGGG.AATCCC	Unfolds G4Qs	Complex of 3 proteins; CTC1 contains the DNA-binding site
hnRNP A1 and UP1	(TTAGGG)n	Unfolds G4Qs	Specifically, nYAGn seq Binds RNA and DNA
BLM	(TTAGGG)n	Unfolds G4Qs (leading strand) in 3′-5′	Needs a ssDNA spacer between G4Q and the replication fork to function Similar to WRN
WRN	(TTAGGG)n	Unfolds G4Qs (lagging strand) in 3′-5′	Similar to BLM
BRCA1	(TTAGGG)n		Binds G4Qs in vitro
Pif1	(TTAGGG)n	Unfolds 5′-3′ direction	
Replication G4BPs			
FANCJ		Unfolds G4Qs in 5′-3′ direction	unwinds both intramolecular and intermolecular G4Qs
Promoter G4BPs			
Sp1	G4 (C) G3 (CC) G5 (C) G4 (TCCCGGC) G4 (CGG) (VEGF) CCCGGGCGGGCGCGAGGGAGGGGAGG (c-KIT) CGGGGCGGGGCGGGGGCGGGGGCG (HRAS)		Binds parallel and antiparallel G4Qs
DHX36	Many sequences	Unfolds G4Qs in 3′-5′	Strong preference for binding parallel DNA and RNA G4Qs over antiparallel; requires trailing 3′ end
Nucleolin	TGGGGAGGGTGGGGAGGGTGGGGAAGG (c-MYC) (GGGGCC)n (HRE)	Stabilizes G4Q to suppress transcription	Preferentially binds parallel G4Qs but can bind both
NM23-H2	TGGGGAGGGTGGGGAGGGTGGGGAAGG (c-MYC)	Unfolds G4Q to enable transcription	
MAZ	TGGGGAGGGTGGGGAGGGTGGGGAAGG (c-MYC) ACAGGGGTGTGGGG (Pur-1) TCGGGTTGCGGGCGCAGGGCACGGGCG and CGGGGCGGGGCGGGGGCGGGGGCG (HRAS) GGGAGGGAGGGAAGGAGGGAGGGAGGGA (KRAS)	Unfolds G4Qs to enable transcription	
PARP1	C3G3CG3CGCGAG3AG4AG2 (c-KIT) TGGGGAGGGTGGGGAGGGTGGGGAAGG (c-MYC) GGGAGGGAGGGAAGGAGGGAGGGAGGGA (KRAS)		Recognizes parallel G4Qs; binding with c-KIT activates PARP1
XPD/XPB		XPD: 5′-3′ direction XPB: 3′-5′ direction	
hnRNP	GGGAGGGAGGGAAGGAGGGAGGGAGGGA (KRAS) GGGGTGGGGCCCTGCGAGGGCGGG (TRA2β)	Unfolds G4Q	
RNA G4BPs			
hnRNP A1	AACGAGGGAGGGAGGGAGAGGGAGAGA- (MMP16) AGCCGGGGGCUGGGCGGGGACCG GGCUUGU (ARPC2)	Unfolds G4Q	
DHX36	Many sequences		
DHX9	Many sequences	Unfolds G4Qs in 3′-5′	Preference for RNA substrates; requires a 3′ single-stranded tail for initial binding
FMRP	GUGUGGAAGGAGUGGCUGGGUUG(sc1)	Stabilizes the G4Q	

The loops of G4Q-forming regions have also been shown to influence the type of G4Q able to form (i.e., parallel vs anti-parallel) (Bugaut and Balasubramanian 2008; Hazel et al. 2004; Tippana et al. 2014). G4BPs appear to have preference for some arrangements over others. For example, DHX36 appears to favor G4Qs with a parallel conformation (but able to bind anti-parallel), and if in fact its binding mode is from the top of the G4Q barrel, this could be reasoned as a less sterically hindered arrangement for this form of binding. Conversely, nucleolin while again favoring parallel G4Qs, has a strong preference for long-looped G4Qs, implying a side-on interaction with the structure (Lago et al. 2017).

Coupling this discussion are the examples of G4BPs that are selective of RNA over DNA and vice versa. For example, DHX36’s selective binding of YY1’s promoter G4Q, but not its mRNA equivalent, suggesting a G4Q backbone interaction is also involved (Huang et al. 2012). It is likely that both loops and core G4Q structural features play some role in providing the appropriate selectivity and specificity needed for the G4BP interaction and subsequent cellular pathway outcome.

## Binding versus functioning

Binding of a G4BP to a G4Q does not equate to functioning. This is demonstrated importantly with the effects of loop modifications on the c-KIT promoter G4Q in complex with PARP1 (Edwards et al. 2020). PARP1 affinity for the G4Q increased as the loop features were removed, however, the complex was no longer able to activate PARP (Edwards et al. 2020). In this study, there was a clear link with the pentanucleotide loop as playing a role in this interaction, as noted above. This also highlights the fact that protein functioning in nature and the energetics of an interaction do not always hold a linear correlation. This is of course an important distinction as we try to design functional assays to support our biophysical claims in in vivo systems.

## Concluding remarks

G4BPs play a central role in many cellular processes, from transcription to translation to genomic stabilization. With the abundance of G4Q sequences present in the cell, it follows that a G4BP must have some selective manner of interacting with its G4Q. Such, binding modes discussed here that may afford selectivity are the top-, groove- and loop-binding interactions. While only a few high-resolution structural examples exist of each, combinations of these modes in these interacting species are apparent. As the field of G4Q-G4BPs continues to evolve—new binding proteins discovered and structures solved, further insights into their functions and cellular pathogenesis will be gained.
